# Misdiagnosis and undiagnosis due to pattern similarity in Chinese medicine: a stochastic simulation study using pattern differentiation algorithm

**DOI:** 10.1186/1749-8546-6-1

**Published:** 2011-01-12

**Authors:** Arthur Sá Ferreira

**Affiliations:** 1 Program of Rehabilitation Science, Centro Universitário Augusto Motta, Av. Paris 72, Bonsucesso, Rio de Janeiro, BR CEP 21041-020, Brazil; 2 Department of Physical Therapy, Universidade Salgado de Oliveira, Rua Marechal Deodoro 263, Niterói, Rio de Janeiro, BR CEP 24030-060, Brazil

## Abstract

**Background:**

Whether pattern similarity causes misdiagnosis and undiagnosis in Chinese medicine is unknown. This study aims to test the effect of pattern similarity and examination methods on diagnostic outcomes of pattern differentiation algorithm (PDA).

**Methods:**

A dataset with 73 *Zangfu *single patterns was used with manifestations according to the Four Examinations, namely inspection (Ip), auscultation and olfaction (AO), inquiry (Iq) and palpation (P). PDA was applied to 100 true positive and 100 true negative manifestation profiles per pattern in simulation. Four runs of simulations were used according to the Four Examinations: Ip, Ip+AO, Ip+AO+Iq and Ip+AO+Iq+P. Three pattern differentiation outcomes were separated, namely correct diagnosis, misdiagnosis and undiagnosis. Outcomes frequencies, dual pattern similarity and pattern-dataset similarity were calculated.

**Results:**

Dual pattern similarity was associated with Four Examinations (gamma = -0.646, *P *< 0.01). Combination of Four Examinations was associated (gamma = -0.618, *P *< 0.01) with decreasing frequencies of pattern differentiation errors, being less influenced by pattern-dataset similarity (Ip: gamma = 0.684; Ip+AO: gamma = 0.660; Ip+AO+Iq: gamma = 0.398; Ip+AO+Iq+P: gamma = 0.286, *P *< 0.01 for all combinations).

**Conclusion:**

Applied in an incremental manner, Four Examinations progressively reduce the association between pattern similarity and pattern differentiation outcome and are recommended to avoid misdiagnosis and undiagnosis due to similarity.

## Background

### Diagnostic process in Western and Chinese medicines

Diagnosis is a process whereby illnesses are recognised and labelled so that appropriate intervention can be taken [1]. In Western medicine, patients' complaints are obtained through both clinical history (inquiry) and physical examination (auscultation, olfaction and palpation) [2,3]. Laboratory tests and images are often necessary for detecting subclinical disturbances or elucidating the ongoing morbid process. Data are interpreted according to the current, biopsychosocial model of health-disease process [4] and hypothetic-deductive reasoning and heuristics are used to establish diagnosis by confirmation of a target hypothesis, rejection of alternative ones or performing differential diagnosis among diagnostic hypotheses [5]. This decision-making is also a pattern recognition process [6], *ie *to diagnose is to identify a stable cluster of possibly concurrent signs and symptoms that are both maximally related to one another and independent of other clusters [7].

In Chinese medicine, diagnosis is also important. Practitioners recognise and label nosological conditions based on inspection (Ip, *wang*), auscultation and olfaction (AO, *wen*), inquiry (Iq, *wen*) and palpation (P, *qie*), also known as the Four Examinations (*Sizhen*). According to traditional literature [8], these methods should be applied in order to enhance recovery of the patients. Manifestations (*ie *signs and symptoms) collected from patients are interpreted using Chinese medicine theories (*eg *eight principles, five phases, vital substances, six channels, four levels, triple burner and *Zangfu*) [9], which were developed on the basis of some observations of Nature [10, 11]. Similar to Western medicine, the collected manifestations are interpreted collectively; however, diagnosis is established through a *pattern differentiation *process whereby a unique, stable manifestation profile is obtained for the identification of a pattern among other diagnostic hypotheses.

*Zangfu *theory is often used to interpret the patient's manifestations, relating the internal organs of the body to its exterior in terms of physiological and philosophical relations. A *Zangfu *single pattern (ZFSP) is characterised by the presence or absence of manifestations depending on aspects such as individual constitution, illness location, stage or severity, collectively known as pattern dynamism [11]. Ancient Chinese medicine literature [8,12-15] is rich in case records, allowing the ready assignment of manifestations related to ZFSP according to the Four Examinations as well as the assignment of new manifestations and identification of contemporary patterns.

Clinically, a patient's manifestation profile is a subset of all possible manifestations characterising the patient's true ZFSP. Therefore, there may be several manifestation profiles that result in the same diagnosis; conversely, a manifestation profile may indicate several ZFSPs.

Patterns, as related to illnesses [16], may be associated or dissociated to other patterns by factors such as: manifestations, relations to tissues, organs and systems, family history and environmental aetiology [10]. Xu Dachun (AD 1693-1771), a Chinese medicine practitioner in the Qing dynasty, stated that '...one may mistakenly confuse the pathocondition of one [illness] with that of the other' [17]. According to Xu, the co-occurrence of manifestations and consequently the amount of shared manifestations between two or more patterns reflects pattern similarity. Pattern similarity introduces errors in the pattern differentiation process as the patient's true pattern may not be properly assigned. Despite its theoretical relevance, the influence of pattern similarity on the accuracy of pattern differentiation is lacking in contemporary scientific literature.

### Types and sources of errors in pattern differentiation process

Three major types of diagnostic errors were identified among Western medicine practitioners, namely no-fault errors, system errors and cognitive errors [18]. Reports of errors for Chinese medicine practitioners are available from ancient literature [8,12-15] including non-skilled practice, misdiagnosis and mistreatment; however, little contemporary literature is available on this subject. Evidence shows that subjectivity of manifestations or limited detection of clinical features is the major causes of unreliable pattern differentiation made by Chinese medicine practitioners [19,20]. Most Western medicine types of errors are applicable to Chinese medicine as well. While diagnostic errors can never be eliminated, they can be minimised through understanding factors related to the pattern differentiation process.

Currently three pattern differentiation outcomes can be distinguished, namely (a) identification of the true pattern (*correct diagnosis*), (b) identification of a pattern that is not the true pattern (*misdiagnosis*) and (c) no identification of pattern at all (*undiagnosis*). Correct diagnosis allows immediate treatment for the patient with proper therapeutic methods. Misdiagnosis affects the selection of specific acupoints and herb combinations [21,22]. Undiagnosis results in delayed diagnosis and treatment, which contradicts the practice of Chinese medicine by 'superior' doctors whose aim is 'to treat those who are not yet ill' [8,12-15].

### Assessment of errors in pattern differentiation process

To test the pattern differentiation process in search for errors, one must ensure that at least the following three conditions are satisfied: (1) patients must accurately report their manifestations, avoiding the no-fault error 'uncertainty regarding the state of the world'; (2) Chinese medicine practitioners must accurately identify signs, avoiding the cognitive errors category 'inadequate knowledge'; and (3) Chinese medicine practitioners must apply objective methods for pattern differentiation according to existing medical theories, avoiding the no-fault error category 'limitations of medical knowledge' [18]. Conditions 2 and 3 may be substantially improved by Chinese medical training [18] as shown in rheumatoid arthritis [23,24] and consequently are possible to achieve in studies with human experts. On the other hand, improvement of condition 1 is limited because it strongly depends on the inherent variability in how patients perceive and describe their health status or their actual symptoms [18,25].

Automatic diagnostic methods are preferable provided that they are accurate, reliable and consistent. Several computational methods for pattern differentiation are available [26-33]. Wang *et al. *[26] did not report accuracy rates for diagnoses but discussed the high dimensionality of patient instances represented by multiple manifestations and diagnostic hypotheses. Their results suggested the use of most frequent attributes to reduce such dimensionality and consequently increase diagnostic accuracy. Zheng and Wu [27] advocated the use of the Four Examinations but did not present any data to validate this recommendation. The authors only described methods to be implemented for an objective assessment of diagnostic with description of a single test case. Yang *et al*. [28] reported an accuracy of 95% after classification of 2000 cases and did not comment on the factors involved in diagnostic errors or their possible types. Huang and Chen [29] also stated that the Four Examinations were necessary correct diagnosis. The authors reported 'high reliable and accurate diagnostic capabilities' in 95% of 50 simulated cases without any description of either how cases were simulated or possible sources and types of error. Liu *et al. *[32] obtained up to 78% accuracy using only the Inquiry method (*n *= 185 manifestations) for identification of multi-patterns (based on 6 ZFSPs) related to coronary heart disease obtained from real cases. For comparison, using the Inquiry method for simulation and identification PDA obtained 89.7% accuracy [30] for 69 ZFSPs and 94.3% [93.9, 94.7] for identification of 73 ZFSPs (obtained as described in the Methods section). While these authors discussed that the frequency of occurrence of manifestations might have affected diagnostic accuracy (since they presented different relations with the main diagnosis), they did not discussed the possible effect of considering other Examinations in the diagnostic accuracy rates. Recently, pattern differentiation algorithm (PDA) was proposed and achieved 94.7% accuracy for ZFSPs using the Four Examinations with sensitivity and specificity of 89.8% and 99.5% respectively [31]. This method allowed testing the impact of different combinations of the Four Examinations and the amount of available information presented by patients on PDA's statistical performance [30,31]. The validation method of PDA used simulation of manifestation profiles, thereby simultaneously overcoming condition 1 and satisfying conditions 2 and 3 as well as allowing the assessment of errors in pattern differentiation process.

The present study aims to investigate the effect of pattern similarity on errors in pattern differentiation. In particular, it aims to separate misdiagnosis from undiagnosis errors associated with pattern similarity. The method is to apply ZFSPs using combinations of the Four Examinations identified with PDA.

## Methods

This study was conducted in the following sequence. Firstly, a stochastic computational simulation based on Monte Carlo method [34,35] was implemented for patient simulation from ZFSP in a dataset. In sequence, simulated manifestation profiles were applied to PDA for automatic pattern differentiation. Pattern similarity was evaluated using objective criteria regarding shared manifestations with other patterns and whole dataset. Pattern differentiation outcomes were categorised in correct diagnosis, misdiagnosis and undiagnosis. Finally, the role of similarity on the diagnostic accuracy was obtained with cross-tables organized by combinations of the Four Examinations. This work followed the Standards for Reporting of Diagnostic Accuracy [36] where applicable to simulation studies.

### Pattern dataset

#### Description

The pattern dataset was expanded for this research following previous works [30,31]. Seventy-three *Zangfu *single patterns (Additional file 1) were listed and all possible manifestations of each pattern *K *(*K *= 1, 2... 73) were assigned separately according to the Four Examinations [9,37]. The total quantity of manifestations describing pattern *K *in the dataset was represented by *N_T,K_*. This quantity *N_T,K _*was derived by counting the absolute quantity of terms in the dataset separated by comma with case-insensitive letters according to the Four Examinations. Manifestations were described specifically including onset ('palpitation in the morning', 'palpitation in the evening'), duration ('acute headache', 'chronic headache'), location ('occipital headache', 'ocular headache') and severity ('dry tongue', 'slightly moist tongue', 'moist tongue'). Manifestations that co-occurred in two or more patterns were assigned with the same term or expression (to increase the accuracy of exact string search algorithm. A total of  539 manifestations was distributed among Ip (n=112, 20.8%; 4 [0-16]), AO (n=42,  7.8%; 0 [0-6]), Iq (n=359, 66.6%; 9 [2-29]) and P (n=26, 4.8%; 2 [0-5]) in the dataset.

#### Dataset quality: intra-pattern and inter-pattern tests

Dataset consistency was computationally tested prior to this study as described previously [31]. Briefly, intra-pattern consistency was obtained through exclusion of repetitions of any manifestation among the Four Examinations that were introduced during manifestation assignment. Inter-pattern consistency was obtained by ensuring that two patterns were not described with the same complete manifestation profile regarding the Four Examinations. In the dataset, for each manifestation there was at least one possible pattern and there was no pattern without manifestations according to the Four Examinations. The complete dataset is available in Portuguese upon request.

### Manifestation profile simulation algorithm

#### Study population

Cases (true positive) and true negative (controls) manifestation profiles were generated by the manifestation profile simulation algorithm (MPSA) described previously [30,31]. The inclusion criterion was the simulation of manifestation profiles using pattern descriptions from the ZFSP dataset. In both simulations, we assumed that the probability of each manifestation in the general population was given and followed a uniformed distribution.

#### Sample size

Sample sizes were estimated from previous results of PDA and equations derived for detecting differences in accuracy tests using receiver operating curves [38]. A minimum sample size of 4,419 manifestation profiles (61 true positive and 61 true negative per pattern) is necessary to detect a 1% difference in accuracy (best accuracy obtained with PDA = 94.7%) [31], with α = 5% (Z_α _= 1.645, one-sided test significance) and β = 90% (Z_β _= 1.28, power of test).

#### Participant recruitment and sampling

Two hundred (100 true positive and 100 true negative) manifestation profiles were prospectively generated for each one of the 73 ZFSPs for the following incremental combinations of the Four Examinations: Ip; Ip+AO; Ip+AO+Iq; Ip+AO+Iq+P. The total sample size was 14,600 per run of simulation (7,300 cases and controls), totaling 58,400 manifestations profiles.

#### Data collection (simulation) of true positive cases

True positive cases of *Zangfu *pattern *K *were simulated by selecting from the dataset a pseudorandom quantity (*N_R,K_*) in the interval (1; *N_T,K_) *among the selected combination of the Four Examinations. Each sorted manifestation was excluded from the set of possible manifestations to prevent multiple occurrences of the same manifestation at the respective simulated case (random sampling method without replacement [39]. This iterative process continued until the *N_R,K _*manifestations were sorted to simulate the manifestation profile.

#### Data collection (simulation) of true negative controls

True negative controls for the same pattern *K *were obtained by sorting *N_R,K _*manifestations from another pattern pseudo-randomly chosen in the dataset after exclusion of pattern *K*. Although the true positive pattern was removed from the dataset, its manifestations that co-occur in other patterns were still present and could be selected to compose a true negative manifestation profile.

#### Missing cases

As it was possible that patterns did not represent manifestations for some of the examination methods, empty manifestation profiles related to these examination methods represented missing cases and were excluded from further analysis.

#### Quality of simulation: consistency between simulated cases and dataset

A new algorithm was implemented for this study to check if all manifestations were used for simulation of manifestations profiles. The algorithm performed a 'reverse engineering' by recreating the dataset from all simulated true positive cases. The algorithm searched among all manifestation profiles simulated for each ZFSP and grouped the manifestations present at least once among the simulated cases into a temporary dataset. After comparison with the original MPSA dataset, the algorithm reported the patterns that were completely simulated (*ie *all manifestations were used for analysis), partially simulated and not used for simulation.

#### Output from MPSA

The MPSA output for each manifestation profile: the name of the simulated pattern *K*; *N_R,K_*; *N_T,K_; *and the manifestations as quoted terms, terms separated by commas. These manifestations were used as inputs for PDA described in the next section.

### Pattern differentiation algorithm

PDA was presented and validated for ZFSP using a criterion based on the amount of explained information [30]. The pseudo-code and the validation of an additional criterion based on the amount of available information were presented [31]. Briefly, the algorithm performed pattern differentiation in a three-stage schema using the same pattern dataset used for simulation of manifestation profiles as follows.

#### Data entry and hypotheses generation

After data entry of manifestations (either by MPSA or a human expert), PDA searched with a combinatorial procedure for quoted terms. Sequentially, a list of candidate patterns was generated with patterns that explain at least one manifestation collected at the exam. Patterns with no manifestations recognized were excluded at this stage.

#### Ranking candidate patterns to obtain diagnostic hypotheses

Candidate patterns were ranked in descending order of *F_%,K _*(the amount of explained information; equation 1), followed by ranking in ascending order of *N_%−cutoff _*(the optimum normalized available information, equation 2):

(1) $$F_{\%,K}=\frac{N_{E,K}}{N_{P}}\times 100\%$$

(2) $$N_{\%-cutoff}=\left(\frac{N_{E,K}}{N_{T,K}}\times 100\%\right)-cutoff$$

where *N_E,K _*is the number of explained manifestations for pattern *K *within the candidate patterns list and *N_P _*is the number of represented manifestations either from simulated profiles or real patients. The optimal value of cutoff in *N_%−cutoff _*was estimated by the same simulation procedure described previously [31], with the current patterns dataset regarding combinations of the Four Examinations. The estimated cutoff values for the dataset of this study were *N_% _*= 51.5% (Ip), *N_% _*= 51.5% (Ip+AO), *N_% _*= 26.5% (Ip+AO+Iq) and *N_% _*= 24.5% (Ip+AO+Iq+P). The resulting ranked list comprised diagnostic hypotheses for consideration during the last stage.

#### Pattern differentiation outcomes

The process was considered successful if PDA found a single pattern *K *among diagnostic hypotheses with the pair (high-unique *F_%,K_*; low-unique *N_%−cutoff_*). Notice that the identified was not necessarily the true pattern, *ie *correct diagnosis and misdiagnosis outcomes respectively. If two or more patterns with equal top-ranked paired values (*F_%,K_; N_%−cutoff_*) were found among diagnostic hypotheses, the process was unsuccessful because differentiation among single patterns was not possible with both explained and available information (undiagnosis outcome). The diagnosis of each manifestation profile was made according to the respective combination of the Four Examinations used to simulate profiles.

#### Output from PDA

PDA output for each tested profile the name of the identified pattern or a message indicating that no pattern was identified at all. This information was used for further classification of the pattern differentiation outcome concerning the reference standard.

### Reference standard

Because cases and controls were simulated for all possible patterns described in the dataset, the output of PDA was compared to the name of the respective simulated pattern. Therefore, in the case of identified patterns, the statistical algorithm checks whether the outputted pattern name matched the simulated one provided in the dataset.

The results of such comparison yielded the diagnostic outcome of PDA, namely correct diagnosis, misdiagnosis and undiagnosis, as explained below. Thus, it was considered the gold-standard method for comparison with the output by PDA.

### Assessment of pattern similarity and diagnostic outcomes for error analysis

A method for co-occurrence of manifestations was implemented based on similarity estimation and computation of pattern differentiation outcome. True negative controls were not used in this analysis since it was necessary to simulate accurate reports of patient's manifestations regarding the true pattern to satisfy condition 1 (see the Background section for details).

#### Computation of dual pattern similarity

Seventy-three patterns on dataset define 2628 (with 73[73-1]/2) unique dual patterns *K_i _*and *K_j _*in the upper triangle of a symmetrical matrix *M_S_*. Each dual pattern was assigned a similarity score *S *defined as the Jaccard coefficient [40-42] (equation 3).

(3) $$S=\frac{F_{ij}}{F_{i}+F_{j}-F_{ij}}$$

where *F_ij _*is the number of manifestations contained in both patterns; *F_i _*and *F_j _*are the number of manifestations contained in either single patterns *K_i _*or *K_j _*members of the dual pattern. *S *is in range [0, 1] indicating no similarity (perfect dissimilarity) and perfect similarity respectively. The lower boundary condition is satisfied by dual patterns that do not share any manifestation (perfectly dissimilar patterns). The upper boundary condition is satisfied by dual patterns which all but one of the manifestations are shared. Perfectly similar patterns are not the upper bound as they describe the same pattern.

#### Computation of pattern-dataset similarity

A measure of similarity between pattern *K *and all other patterns in dataset were also calculated, besides in a dual pattern basis. Such coefficient must, for the same absolute amount of shared manifestations, result in the same similarity value if calculated with equation 3. Thus, it was proposed a variant of Jaccard coefficient S* defined as follows (equation 4).

(4) $$S*=\frac{F_{id}}{2F_{i}-F_{id}}$$

where *F_id _*is the number of manifestations contained in both single pattern *K *and the whole dataset (excluding pattern *K *itself). The replacement of *F_j _*by *F_i _*is necessary to achieve the upper limit value of similarity when all manifestations are shared: if *F_id _*= *F_i _*then S* = *F_id_*/(2*F_id _*- *F_id_*) = 1. Moreover, when all manifestations of pattern *K *are exclusive to such pattern (i.e., pathognomonic) one have *F_id _*= 0 and S* = 0. Thus, this coefficient of association reflects the amount of shared manifestations of pattern *K *that can be found in the dataset after its exclusion.

#### Computation of pattern differentiation outcomes

The comparison of diagnostic outcomes would result in a 2 × 2 contingency table where cases and controls are classified as being or not with a particular condition [43]. For this study, the 'wrong' outcomes (false positive and false negative profiles) were separated into two specific conditions (misdiagnosed and undiagnosed patterns). The following conditions resulted from comparison between simulated and identified patterns:

(1) Cases: *If *'identified pattern' = 'simulated pattern' *then *outcome = 'correct diagnosis'; *else*

(2) *If *'identified pattern'≠'simulated pattern' *then *outcome = 'misdiagnosis'; *else*

(3) *If *'identified pattern' = [ ] *then *outcome = 'undiagnosis'; *end*

(4) Controls: *If *'identified pattern'≠'simulated pattern' *then *outcome = 'correct diagnosis'; *else*

5) *If *'identified pattern' = 'simulated pattern' *then *outcome = 'misdiagnosis'; *else*

6) *If *'identified pattern' = [ ] *then *outcome = 'undiagnosis'; *end*.

### Statistical analysis

#### Choice of variables and statistical methods

Since both coefficients of similarity *S *and S* are continuous variables and represent the 'strength of association' between patterns, they were categorized as an association measure (ordinal variable) [44]: 0.00 (no similarity); 0.01 to 0.20 (negligible); 0.21 to 0.40 (weak); 0.41 to 0.70 (moderate); 0.71 to 0.99 (strong); 1.00 (perfect similarity). As the Four Examinations were applied as a cumulative procedure with recommended order of application [8], it was also considered as an ordinal variable. Finally, pattern differentiation outcome was considered as an ordinal variable since the consequences of the outcomes (*ie *correct, mistaken, and absent) regarding both treatment and prognosis are intrinsically worse in this particular order. Thus, two ordinal measures of association were used to evaluate whether there was monotonic linear relations in cross-tables: Goodman-Kruskal γ [45,46] and the squared value of its variant γ*^2 ^[47]. Coefficient γ is in range [-1, 1], indicating an exact negative relationship, and an exact positive relationship respectively. The coefficient γ*^2 ^is in range [0, 1] indicating the proportional-reduction-in-variation of one variable when knowing the other one (R^2^-like coefficient). Statistical significance was considered for *P *< 0.05.

#### Association between the Four Examinations and dual pattern similarity

A cross-table was built by simultaneous classification of dual patterns into the categories of similarity *S *and according to the cumulative combinations of the Four Examinations. The null hypothesis was that dual pattern similarity and the Four Examinations were independent variables.

#### Association between the Four Examinations and pattern differentiation outcome

A cross-table was generated by simultaneous classification of simulated cases by pattern differentiation outcome and cumulative combination of examination methods. The null hypothesis was that pattern differentiation outcome and the Four Examinations were independent variables.

#### Association between pattern-dataset similarity and pattern differentiation outcome, grouped by the Four Examinations

A cross-table was generated from pattern-dataset similarity S* and pattern differentiation outcomes grouped by cumulative combination of Four Examinations. The null hypothesis was that pattern similarity and pattern differentiation outcome were independent variables.

#### Test reproducibility

Calculations of reference standard reproducibility were not performed since both true positive and true negative profiles were always generated from the same dataset.

#### Blinding

No user intervention was required during the entire process (simulation of manifestation profiles; cutoff-estimation for *N_%_*; pattern identification with *F_% _*and *N_%-cutoff _*of simulated cases; and statistical analysis). Additionally, MPSA and PDA are composed of independent algorithmic codes (*ie *there is no code sharing), so the results of the identification were blinded to the simulation parameters.

#### Computational resources

All algorithms were implemented in LabVIEW 8.0 (National Instruments, USA) and executed on a 2.26 GHz Intel^® ^Core 2 Duo microprocessor with 2.00 GB RAM running Windows 7 (Microsoft Corporation, USA). Screenshots of the implementations of both MPSA and PDA are presented in the additional files 2 and 3, respectively.

## Results

### Study flowchart and simulation quality

**Figure 1:**
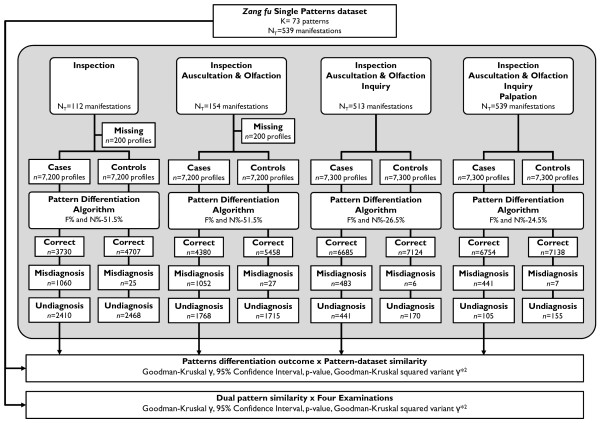
**Flowchart of the simulation study for investigation of pattern differentiation errors**. Departing from *Zangfu *single patterns dataset, manifestation profiles were simulated according to the combination of examination methods. Cases (true positive) manifestation profiles were tested with criteria *F_%,K _*and *N_%-cutoff_*. Pattern differentiation outcomes (correct, misdiagnosis and undiagnosis) were categorized for analysis of association with pattern similarity and the Four Examinations.

The flowchart describing the simulation study is presented in Figure 1. One hundred of 7300 (1.4%) simulated cases were excluded from both Ip and Ip+AO examination methods due to the absence of manifestations in one pattern for those respective examination methods in the dataset. As for the Ip+AO+Iq and Ip+AO+Iq+P runs, all patterns in dataset were fully recreated from the simulated manifestation profiles.

### Four Examinations and dual pattern similarity: intrinsic similarity

The cross-table showing dual pattern frequencies classified by categories of similarity and the cumulative combination of the Four Examinations is presented in Table 1.There was a negligibly, significant association (γ = 0.192, 95% CI = [0.165, 0.219], *P *< 0.01; γ*^2 ^≈ 2%) of dual pattern similarity and combinations of the Four Examinations; however, if the analysis is restricted to those dual patterns that present similarity (*ie *for which *S *> 0), that is if the first column in Table 1 is removed, clearly a stronger association value was obtained (γ = -0.646, 95% CI = [-0.688, 0.604], p < 0.01), which corresponds to a proportional-reduction-in-variation of γ*^2 ^≈ 24%. This result indicates that dual pattern similarity is moderately associated with Four Examinations, with decreasing dual pattern similarity as the Four Examinations were cumulatively grouped.

**Table 1:** Cross-table of dual patterns classified simultaneously by categories of dual pattern similarity and the incremental combination of the Four Examinations.

Four Examinations	Dual pattern similarity, S						Total
	No similarity	Negligible	Weak	Moderate	Strong	Perfect	
Ip	1708	632	220	57	6	5	2628
Ip+AO	1654	748	182	37	2	5	2628
Ip+AO+Iq	1339	1253	32	3	1	0	2628
Ip+AO+Iq+p	1088	1480	54	5	1	0	2628

Ip = Inspection; AO = Auscultation and Olfaction; Iq = Inquiry; P = Palpation.

For S≥: γ = 0.192, 95% CI = [0.165, 0.219], *P *< 0.01; γ*^2 ^≈ 2%.

For S>: γ = -0.646, 95% CI = [-0.688, -0.604], *P *< 0.01; γ*^2 ^≈ 24%.

### Four Examinations and pattern differentiation outcome: types of errors

The cross-table showing pattern differentiation outcome frequencies grouped by the incremental combination of the Four Examinations are presented in Table 2. Concerning true positive cases, the use of the Four Examinations resulted in the highest frequency of correct diagnosis (*n *= 6754), followed by three (Ip+AO+Iq, *n *= 6685), two (Ip+AO, *n *= 4380) and single examination methods (Ip, *n *= 3730). The Four Examinations resulted in the lowest rate of misdiagnosis and undiagnosis (*n *= 441 and *n *= 105 respectively), followed by three (Ip+AO+Iq, *n *= 483 and *n *= 132 respectively), two (Ip+AO, *n *= 1052 and *n *= 1768 respectively) and single examination methods (Ip, *n *= 1060 and *n *= 2410 respectively). There was a significant association (γ = -0.618, 95% CI = [-0.631, -0.606], *P *< 0.01; γ*^2 ^≈ 21%) between pattern differentiation outcome and the Four Examinations, indicating that cumulative application of the Four Examinations is moderately associated with decreasing frequencies of pattern differentiation errors (misdiagnosis and undiagnosis, in this order) and increasing frequencies of correct diagnosis outcome.

**Table 2:** Cross-table of simulated cases and controls classified simultaneously by pattern differentiation outcome and the incremental combination of the Four Examinations

	Pattern differentiation outcome			Missing	Total
Four Examinations	Correct diagnosis	Misdiagnosis	Undiagnosis		
True positive					
Ip	3730	1060	2410	100	7300
Ip+AO	4380	1052	1768	100	7300
Ip+AO+Iq	6685	483	132	0	7300
Ip+AO+Iq+p	6754	441	105	0	7300
Total (TP)	21549	3036	4415	200	29200
					
True negative					
Ip	4707	25	2468	100	7300
Ip+AO	5458	27	1715	100	7300
Ip+AO+Iq	7124	6	170	0	7300
Ip+AO+Iq+p	7138	7	155	0	7300
Total (TN)	24427	65	4508	200	29200
					
Total (TP+TN)	45976	3101	8923	400	58400

For TP: γ = -0.618, 95% CI = [-0.631, -0.606], *P *< 0.01; γ*^2 ^≈ 21%

For TN: γ = -0.709, 95% CI = [-0.722, -0.695], *P *< 0.01; γ*^2 ^≈ 29%

Ip = Inspection; AO = Auscultation and Olfaction; Iq = Inquiry; P = Palpation; TP = true positive cases; TN = true negative controls

Note: Missing cases were due to the absence of the manifestations describing the inspection method. These values were not considered for statistical analysis.

As expected, the same effect was observed among true negative controls. Strong, significant association value (γ = -0.709, 95% CI = [-0.722, -0.695], *P *< 0.01; γ*^2 ^≈ 29%) was found between pattern differentiation outcome and Four Examinations. Incremental application of the Four Examinations was also associated with decreasing frequencies of pattern differentiation errors.

### Effects of pattern-dataset similarity on pattern differentiation errors

The cross-table with pattern-dataset similarity and pattern differentiation outcomes is presented in Table 3, grouped by the Four Examinations. There was a significant association between pattern-dataset similarity and pattern differentiation outcome within each tested combination of the Four Examinations, indicating that an increase in similarity is accompanied by an increase in misidentification and no identification at all and consequently a decrease in correct pattern identification. Such effect was less pronounced when cumulative combination of the Four Examinations were applied, as indicated by a decrease in the association value from moderate weak (Ip : γ = 0.684, 95% CI = [0.660, 0.708], γ*^2 ^≈ 27%; Ip + AO: γ = 0.660, 95% CI = [0.634, 0.686], γ*^2 ^≈ 25%; Ip + AO + Iq: γ = 0.398, 95% CI = [0.339, 0.458], γ*^2 ^≈ 8%; Ip + AO + Iq + P: γ = 0.286, 95% CI = [0.217, 0.355], γ*^2 ^≈ 4%).

**Table 3:** Cross-table of true positive cases classified simultaneously by categories of pattern-dataset similarity and pattern differentiation outcome grouped by incremental combination of the Four Examinations

	Pattern-dataset similarity, S*						Total
Outcomes per Examination	No similarity	Negligible	Weak	Moderate	Strong	Perfect	
Ip							7300
Correct diagnosis	100	100	562	943	586	1439	3730
Misdiagnosis	0	0	18	132	109	801	1060
Undiagnosis	0	0	20	225	105	2060	2410
Missing	-	-	-	-		-	100
							
Ip+AO							7300
Correct diagnosis	100	200	369	1283	760	1668	4380
Misdiagnosis	0	0	15	164	761	712	1652
Undiagnosis	0	0	16	153	79	1520	1768
Missing	-	-	-	-		-	100
							
Ip+AO+Iq							7300
Correct diagnosis	0	100	1048	3638	1462	437	6685
Misdiagnosis	0	0	51	200	107	125	483
Undiagnosis	0	0	1	62	31	38	132
Missing	-	-	-	-		-	0
							
Ip+AO+Iq+p							7300
Correct diagnosis	0	0	671	3839	1840	404	6754
Misdiagnosis	0	0	22	205	133	81	441
Undiagnosis	0	0	7	56	27	15	105
Missing	-	-	-	-		-	0

For Ip: γ = 0.684, 95% CI = [0.660, 0.708], *P *< 0.01; γ*^2 ^≈ 27%

For Ip+AO: γ = 0.660, 95% CI = [0.634, 0.686], *P *< 0.01; γ*^2 ^≈ 25%

For Ip+AO+Iq: γ = 0.398, 95% CI = [0.339, 0.458], *P *< 0.01; γ*^2 ^≈ 8%

For Ip+AO+Iq+p: γ = 0.286, 95% CI = [0.217; 0.355], *P *< 0.01; γ*^2 ^≈ 4%

Ip = Inspection; AO = Auscultation and Olfaction; Iq = Inquiry; P = Palpation.

Note: Missing cases were due to the absence of the manifestations describing the inspection method. These values were not considered for statistical analysis.

## Discussion

This study investigated the effect of pattern similarity on pattern differentiation errors regarding the Four Examinations. The main results include: (1) two types of pattern differentiation errors were distinguished within PDA, namely misdiagnosis and undiagnosis; (2) pattern differentiation errors were affected by either dual pattern and pattern-dataset similarities and (3) misdiagnosis and undiagnosis frequencies due to pattern similarity were minimised under cumulative use of individual Examination methods.

### Distinction of pattern differentiation errors: misdiagnosis and undiagnosis

The distinction of types of wrong outcomes is relevant since methodological approaches for their correction are different. While errors are expected to occur, this is the first study to investigate types of error in the pattern differentiation process. Recent reviews and articles on computational methods applied to Chinese medicine lack evidence for sources of diagnostic errors [48,49]. Several methodological flaws were described by these reviews regarding previous studies in diagnostic accuracy [26-30,32,33]. We could not test them for sources of errors because: the algorithm was not sufficiently described [27]; the algorithms were validated using real cases [26,28,29,32] (subjected to missing or inappropriate reference standards [33]); the algorithm was validated using simulated cases but under-specified procedure that does not allow reproduction.

Previous studies with PDA did not investigate types of errors in pattern differentiation or its association with pattern similarity. Accuracies in range 70.7% to 93.2% were obtained with cumulative combination of the Four Examinations [30]. In a subsequent work [31], the observed accuracies increased to range 74.3% to 94.7% with the cumulative Examinations after insertion of the available information as a new objective criterion for pattern differentiation; however, in these two studies, the diagnostic outcome was classified only as successful or unsuccessful (2 × 2 contingency table), making no distinction of different error types among unsuccessfully outcomes. The distinction of error types in this study was possible due to the change in nature of manifestation profiles from the above-mentioned studies. In the present study, true negative controls were any other true ZFSP that was not its true positive counterpart, and not just random manifestations from all patterns in dataset as in those studies [30,31]. This modification expanded the interpretation of false negative *K_i _*cases from one wide option ('it can be any other pattern *K_j_*, no pattern at all, or it was not possible to uniquely identify any pattern *K*') into two separate options ('it is pattern *K_j_*' or 'it was not possible to uniquely identify any pattern in dataset'). With this true condition made known a priori it was possible to distinguish misidentification from no identification among unsuccessful outcomes as described in the Methods section. Nevertheless, the methods described in the present study may be used to test pattern differentiation outcomes from any other system (either automatic or 'human') provided that true positive and true negative manifestations profiles have their true diagnosis known or, at least, assumed.

### Effect of pattern similarity on pattern differentiation errors

Although pattern similarity is an expected factor influencing diagnostic outcomes, another original contribution of the present study is the provision of an estimate of the extent of possible pattern differentiation errors due to pattern similarity regarding the Four Examinations. Dual pattern similarity has moderate, statistically significant effect on pattern differentiation outcome (Table 2). As stated above, current literature on this topic lacks evidence of pattern differentiation errors as well as their sources and relative contribution to total error rates [26-29]. Previous studies with PDA explored diagnostic accuracies under different scenarios: (1) the individual and cumulative use of Four Examinations [30]; and (2) the effect of available information (*ie *manifestations) on diagnostic accuracy [31]. Those results showed that both the Four Examinations and limited available information affect undesirable outcomes rates.

### Pattern differentiation errors due to pattern similarity are minimized under Four Examinations

The results of the present study show that cumulative application of the Four Examinations progressively reduced the strength of significant association between pattern similarity and diagnostic errors (from γ = 0.684 to γ = 0.286; *P *< 0.01 for all tested combinations). Perfect dissimilar dual patterns were not found in dataset until Inspection was not included for pattern differentiation (Table 2). The highest decrease in explained variation between pattern differentiation outcome and similarity was observed when Inquiry was added to the examination procedure (Ip + AO: γ*^2 ^≈ 25%; Ip + AO + Iq: γ*^2 ^≈8%, Table 3). While all examination methods provided dissimilar manifestations, the Inquiry method introduced most of the dissimilarity among patterns in dataset, which in turn resulted in increased correct diagnosis frequencies. Thus, the Inspection may be considered as the best single Examination method to avoid misdiagnosis and undiagnosis due to similarity because it introduced most of the dissimilarity among patterns. This effect was also observed in Western medicine [2,3], where medical history provided enough information to make a correct diagnosis of a specific illness and the other methods were instrumental in excluding diagnostic hypotheses and in increasing the practitioners' confidence in their diagnoses. Because of the usefulness of the Inquiry examination, we suggest that more time should be devoted to improving history-taking skills during clinical training.

Some criticism may arise from the 'particular order' of application of Examination methods. As a corollary of the holistic approach of Chinese medicine, the order in which Examination methods are applied does not change the pattern differentiation outcome. Assuming that practitioners always use the Four Examinations and are successful in this task, they conclude their screening procedure with the same manifestation profile no matter the applied order. Also, neither PDA nor any other algorithm for pattern differentiation discussed [26-31] assumes manifestations are given in a particular order, *ie *all manifestations are considered collectively. This must not be confused with the timeline of onset of manifestations; when at screening, the patient presents simultaneously all manifestations. Although each Examination contributes differently for reducing pattern differentiation errors, it seems that the order in which the Four Examinations are used is just a matter of keeping a rigid routine to ensure that every aspect of screening was performed.

### Perspective for reducing errors due to pattern similarity and consequences of undesirable outcomes in clinical practice

Pattern similarity is intrinsic to Chinese medical knowledge (Table 1). Consequently, continued research is necessary for discovery of strategies for dealing with similarity as a confounding factor. The undiagnosis outcome means that no pattern was uniquely found based on PDA's criteria while misdiagnosis outcome represents the selection of a wrong pattern. In both cases, the correct pattern was always cited as a diagnostic hypothesis due to the algorithmic search strategy. Thus, there is a perspective for further reducing undesirable outcomes.

In case of undiagnosis, the simplest approach would be to make PDA alert the expert practitioner and request manual selection of a pattern from the list of diagnostic hypotheses. Alternatively, the practitioner may choose another Examination method when PDA left a ZSFP undiagnosed. The latter approach is preferable to the former since it does not rely on human intervention for decision-making. The increase in explained variation of each tested combination of Examinations observed in this study suggests that investigations (whether single Examinations or not) are capable of identification of manifestations profiles undiagnosed with the Four Examinations. This is in accordance with the traditional literature. Zhang Zhongjing (early third century) and Sun Simiao (AD 581-682) emphasized the application of single Examinations, concerning their relevance for prognosis: Ip, AO and P [50]. Huang Fumi (AD 215-282) quoted the *Neijing *describing Palpation as 'formal diagnosis' and stated that it might provide a clear picture of the patient [8].

In a real case, if a patient is still left undiagnosed, it is necessary to observe how the pattern evolves. Undiagnosed ZFSPs may worsen and/or transmit through the *Zangfu *system, being more apparent or with more information when compared to the initial unbalanced health status thereby increasing the probability of an accurate diagnosis [31].

Misdiagnosed manifestation profiles are more difficult to resolve than undiagnosed ones because a (wrong) pattern was identified. While the true pattern is known in simulated profiles, this does not hold true for real cases and consequently it is impossible to know in advance when another criterion is necessary; however, some insights may be found in Table 1 where the majority of patterns are dissimilar in a dual pattern analysis. The no-shared manifestations of dissimilar dual patterns guarantees a correct diagnosis in every case since all possible manifestation profiles for pattern *K_i _*will not recall pattern *K_j _*to compose the diagnostic hypotheses. Despite the overall reduction in occurrence of dissimilar dual patterns from Ip to Ip+AO+Iq+P (range 1708-1088 respectively), it is still possible to explore the potential of 'almost pathognomonic' manifestations with negligible and weak dual patterns similarity. For instance, the selection of manifestations was reported to either increase or reduce the diagnostic accuracy of chronic gastritis in individuals with *Helicobacter Pylori *[51]. These highly selective manifestations may be used as 'weight' for occurrence of manifestations or retesting identified patterns.

Another approach for reducing of misdiagnosis is to investigate the consequences of the outcome for intervention. In theory, misdiagnosed patterns should have their therapeutic methods compared to those from the true pattern. If the therapeutic methods are not significantly different (as seen in rheumatoid arthritis [23] and frequent headache [21]), then the patients will not be severely mistreated. In such a case, it may be argued if a correct diagnosis should be achieved in every case where the therapeutic methods are not significantly different. Despite the consideration of acupuncture as a low-risk procedure [52,53], single (*danfang*) and composite herbs (*fufang*) prescriptions are associated with side-effects such as kidney failure [54] and cancer [55]; however, since those therapeutic interventions are frequently associated [37], we suggest the comparison of therapeutic methods as the next step before attempting to use other criterion.

### Methodological considerations

#### Dataset content quality and external validity

The constructed dataset seems to be sufficient for an exploratory analysis on diagnosis of ZFSPs. Literature on standardization of terms and expressions in Chinese medicine report 103 terms related to inspection, 27 to auscultation and olfaction, 203 to inquiry and 80 to palpation, totaling 413 terms or expressions [56]. Moreover, notice that not all terms presented in such literature are clinical manifestations. While such standardization does not intend to be exhaustive, its quantity reflects an expected amount of information to be incorporated in a pattern dataset. The collected manifestations from literature [9,37] resulted in 539 items, approximately 30% of additional information. Thus, compared to World Health Organization standards, the content of the pattern dataset was considered adequate for simulation of ZFSPs; however, it must be emphasized that the dataset used in this study does not intend to contain a definitive description of those studied patterns. The proposed methodology is applicable to any dataset with such information, both theoretical (collected from books) or real patients. In the last case, however, some criticism about the 'true' diagnosis may appear because the known diagnosis may be biased.

#### Consistency between simulated cases and dataset

Results concerning the reconstruction of dataset from all simulated cases reveal that all manifestations were used in all tested combinations of examination methods. While there is no formula specifying the exact number of simulations needed in stochastic simulation studies, it is considered that this number should increase with the amount of information of patterns to reduce simulation variability in the result [57]. Variability arises when manifestations are not considered in simulated cases but do occur in a real sample. Moreover, there is no guarantee that all manifestations are present in a real sample. The absolute consistency found in the present study does not mean that all possible manifestation profiles were tested for each pattern but that at least all manifestations were considered once for analysis. Finally, the equation designed to real cases can be used in simulated ones provided that the absolute consistency between original and recreated datasets is proved. This is an important issue related to the quality control in this study and should not be omitted in other simulations studies were pattern differentiation outcomes are assessed.

## Conclusion

Pattern similarity is moderately associated with pattern differentiation outcome. The traditional combination of the Four Examinations, applied in an incremental manner, progressively reduces the association between pattern similarity and pattern differentiation outcome and is recommended for avoiding misdiagnosis and undiagnosis due to similarity.

## Abbreviations

Ip: inspection; AO: auscultation and olfaction; Iq: inquiry; P: palpation; ZFSP: *Zang-fu *single pattern; PDA: pattern differentiation algorithm; MPSA: manifestation profile simulation algorithm; *K*: single pattern from dataset; *N_T,K_*: quantity of manifestations describing pattern *K *in dataset; *N_R,K_*: quantity of randomly selected manifestations of pattern *K*; *F_%,K_*: proportion of explained information of pattern k from clinical history; *N_%-cutoff_*: proportion of optimized available information of pattern *K *in dataset; *N_E,K_*: quantity of explained manifestations of pattern *K*; *N_P_*: quantity of presented manifestations on the clinical history; *S*: dual pattern similarity; S*: pattern-dataset similarity; ≈: approximately (numeric values rounded to the closest integer value).

## Competing interests

The authors declare that they have no competing interests.

## Authors' contributions

The author performed the study, wrote the manuscript and approved the final version of the manuscript.

## Supplementary Material

Additional file 1 **Seventy-three (73) *Zangfu *single patterns described in the dataset**. This table lists the *Zangfu *single patterns described in the dataset. Click here for file

Additional file 2 **Manifestation profile simulation algorithm**. This file presents screenshots with the source code of the algorithms for simulation of manifestations. Click here for file

Additional file 3 **Pattern differentiation algorithm**. This file presents screenshots with the source code of the algorithms for pattern differentiation. Click here for file
