# Supplementary material for: Misdiagnosis and undiagnosis due to pattern similarity in Chinese medicine: a stochastic simulation study using pattern differentiation algorithm
Source: Chin Med. 2011 Jan 12;6:1. doi: 10.1186/1749-8546-6-1 (PMC3037949; doi:10.1186/1749-8546-6-1)
Supplement: Additional file 1 — Seventy-three (73) Zangfu single patterns described in the dataset. This table lists the Zangfu single patterns described in the dataset. [file 1749-8546-6-1-S1.DOC]

Seventy-three (73) Zangfu single patterns described in the dataset

| 1 | Bladder deficient and Cold | 38 | Kidneys failing to receive Qi |
| --- | --- | --- | --- |
| 2 | Cold invading the Large Intestine | 39 | Large Intestine Dryness |
| 3 | Cold invading the Stomach | 40 | Liver-Blood deficiency |
| 4 | Collapse of Large Intestine | 41 | Liver-Blood stasis |
| 5 | Damp-Cold in the Bladder | 42 | Liver-Fire and Gall-Bladder-Fire |
| 6 | Damp-Cold invading the Spleen | 43 | Liver-Fire blazing upwards |
| 7 | Damp-Heat in Liver and Gall-Bladder | 44 | Liver-Qi stagnant |
| 8 | Damp-Heat in the Bladder | 45 | Liver-Wind agitating within, Deficient Liver-Blood causing Wind |
| 9 | Damp-Heat in the Gall-Bladder | 46 | Liver-Wind agitating within, Extreme Heat generating Wind |
| 10 | Damp-Heat in the Large Intestine | 47 | Liver-Wind agitating within, Liver-Yang rising causing Wind |
| 11 | Damp-Heat invading the Spleen | 48 | Liver-Yang rising |
| 12 | Damp-Heat invading the Spleen and Stomach | 49 | Liver-Yin deficiency |
| 13 | Full-Heat in the Small Intestine | 50 | Lung Dryness |
| 14 | Gall-Bladder deficient | 51 | Lung-Qi deficiency |
| 15 | Heart-Blood deficiency | 52 | Lung-Qi obstruction |
| 16 | Heart-Blood stasis | 53 | Lung-Yin deficiency |
| 17 | Heart-Fire blazing | 54 | Phlegm misting the Mind |
| 18 | Heart-Qi deficiency | 55 | Phlegm-Damp obstructing the Lungs |
| 19 | Heart-Yang collapse | 56 | Phlegm-Fire harassing the Heart |
| 20 | Heart-Yang deficiency | 57 | Phlegm-Fluids obstructing the Lungs |
| 21 | Heart-Yin deficiency | 58 | Phlegm-Heat obstructing the Lungs |
| 22 | Heat in the Large Intestine | 59 | Retention of Food in the Stomach |
| 23 | Heat obstructing the Large Intestine | 60 | Small Intestine deficient and cold |
| 24 | Infestation of worms in the Small Intestine | 61 | Small Intestine Qi pain |
| 25 | Invasion of Large Intestine by Cold | 62 | Small Intestine Qi tied |
| 26 | Invasion of Lungs by Wind-Cold | 63 | Spleen not controlling Blood |
| 27 | Invasion of Lungs by Wind-Dryness | 64 | Spleen-Qi deficiency |
| 28 | Invasion of Lungs by Wind-Heat | 65 | Spleen-Qi sinking |
| 29 | Invasion of Lungs by Wind-Water | 66 | Spleen-Yang deficiency |
| 30 | Kidney-Essence deficiency | 67 | Stagnation of Cold in the Liver channel |
| 31 | Kidney-Qi deficiency | 68 | Stasis of Blood in the Stomach |
| 32 | Kidney-Qi not firm | 69 | Stomach deficient and cold |
| 33 | Kidney-Yang deficiency | 70 | Stomach-Fire |
| 34 | Kidney-Yang deficiency, Water overflowing to the Heart | 71 | Stomach-Qi deficiency |
| 35 | Kidney-Yang deficiency, Water overflowing to the Lungs | 72 | Stomach-Qi rebelling upwards |
| 36 | Kidney-Yin deficiency | 73 | Stomach-Yin deficiency |
| 37 | Kidney-Yin deficiency, Empty-Fire blazing |  |  |
