# Supplementary material for: Misdiagnosis and undiagnosis due to pattern similarity in Chinese medicine: a stochastic simulation study using pattern differentiation algorithm
Source: Chin Med. 2011 Jan 12;6:1. doi: 10.1186/1749-8546-6-1 (PMC3037949; doi:10.1186/1749-8546-6-1)
Supplement: Additional file 2 — Manifestation profile simulation algorithm. This file presents screenshots with the source code of the algorithms for simulation of manifestations. [file 1749-8546-6-1-S2.PDF]

Manifestation Profiles Simulation Algorithm - MPSA.vi

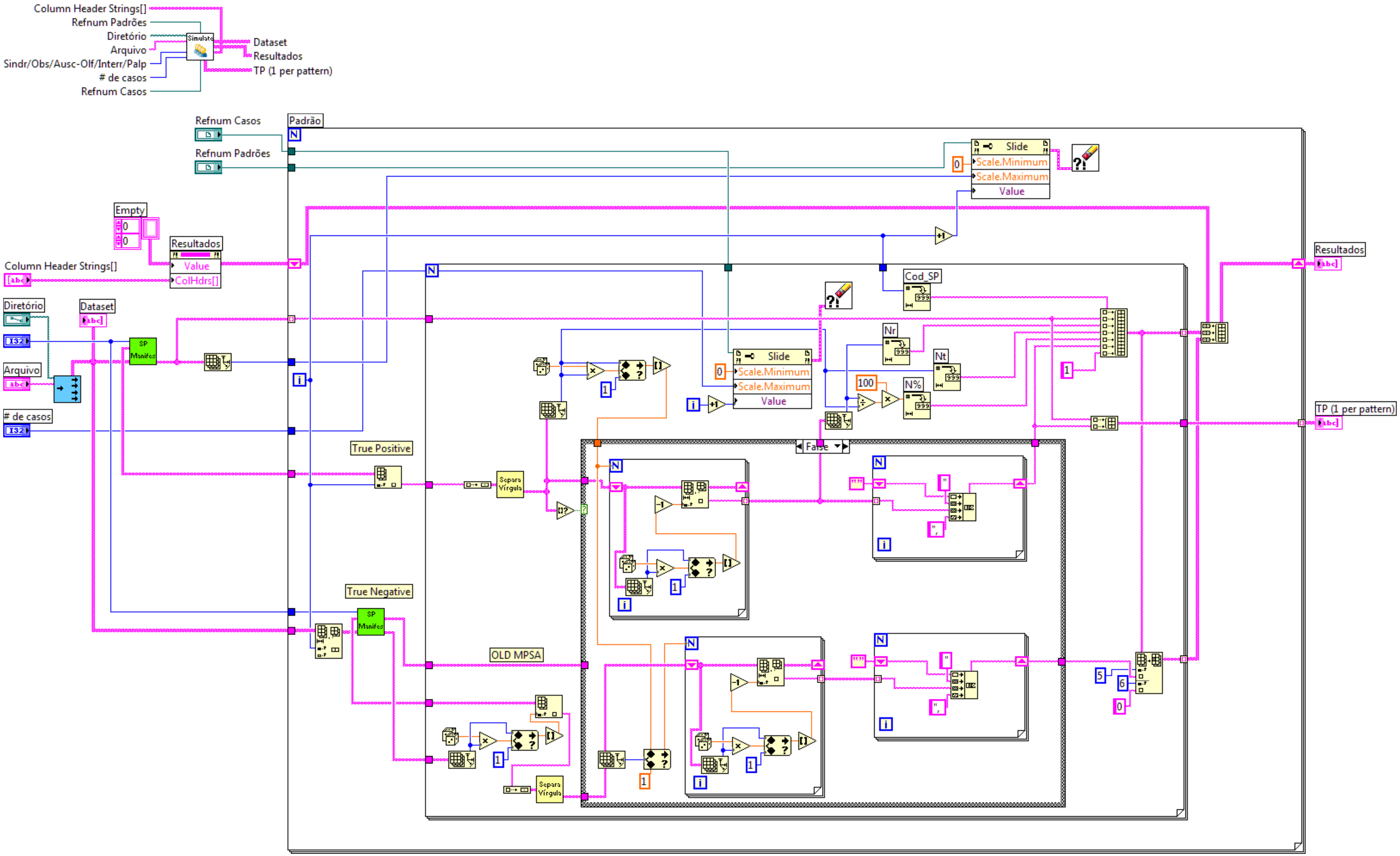

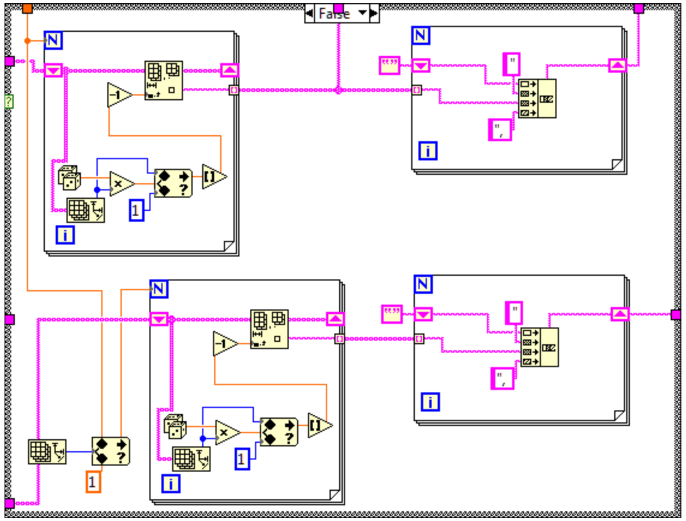

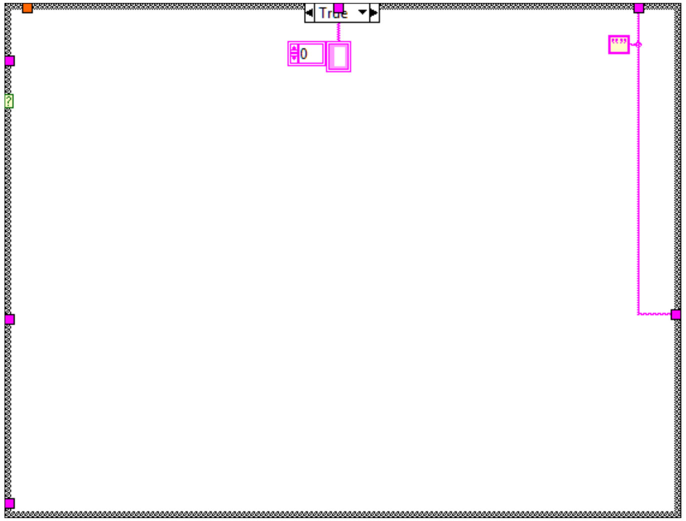

Concatenar database.vi

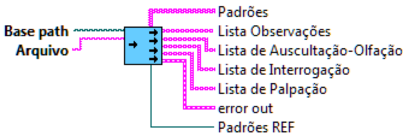

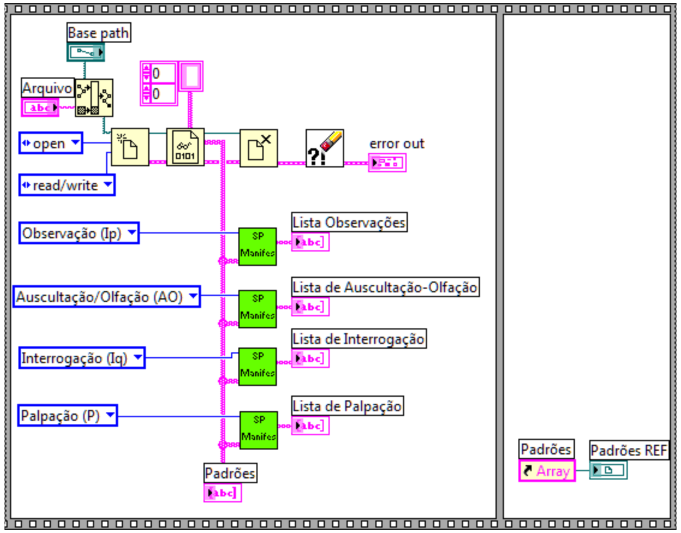

Clear Errors.vi

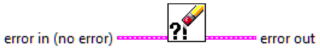

The error in value is not passed to the error out indicator.

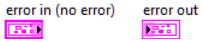

SP - Manifestações.vi

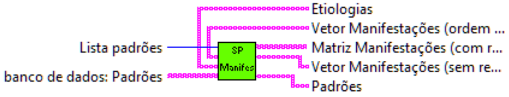

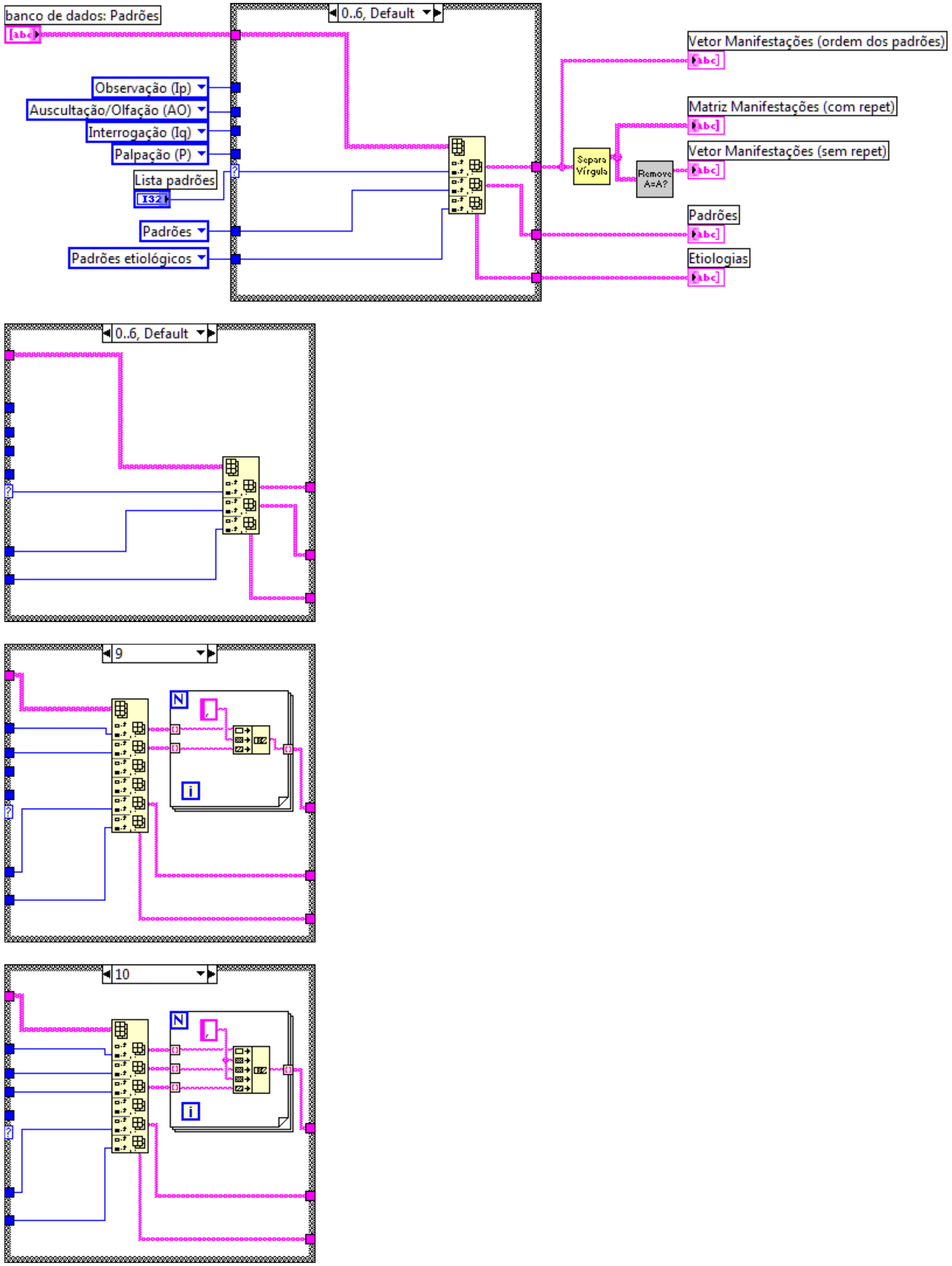
banco de dados: Padrões
0..6, Default
Observação (Ip)
Auscultação/Olfação (AO)
Interrogação (Iq)
Palpação (P)
Lista padrões
I32
Padrões
Padrões etiológicos
Separa Vírgula
Remove A=A?
Vetor Manifestações (ordem dos padrões)
Matriz Manifestações (com repet)
Vetor Manifestações (sem repet)
Padrões
Etiologias
0..6, Default
9
N
i
10
N
i

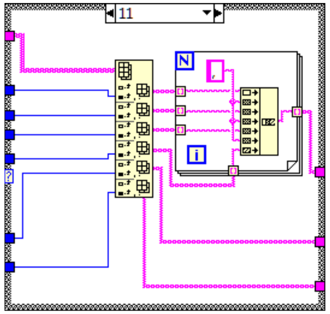

Separa entre vírgulas.vi

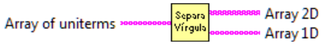

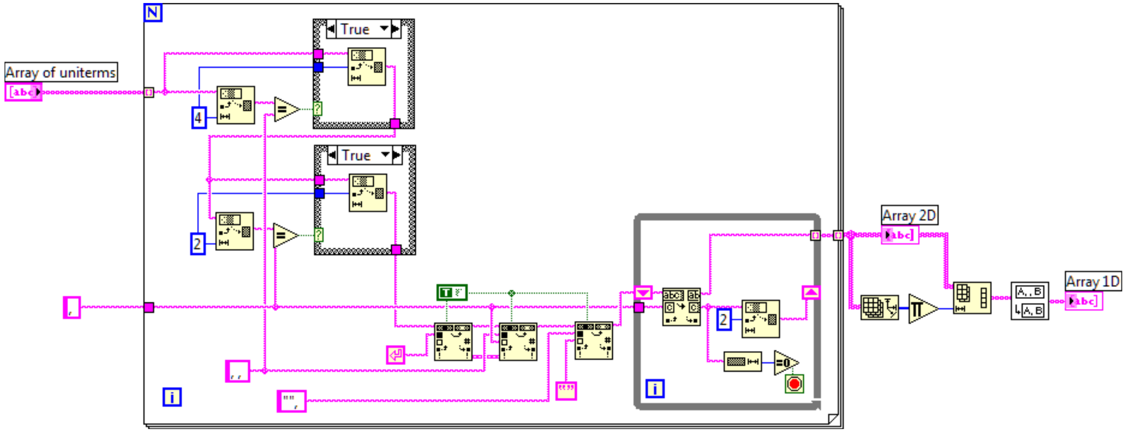

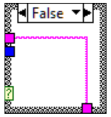

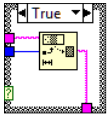

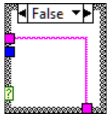

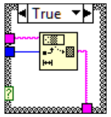

Remove espaços em branco.vi

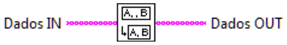

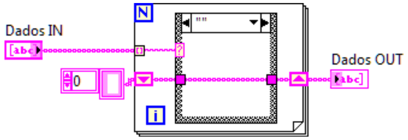

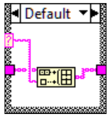

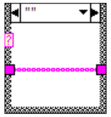

Remove termos repetidos.vi

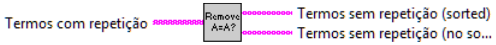

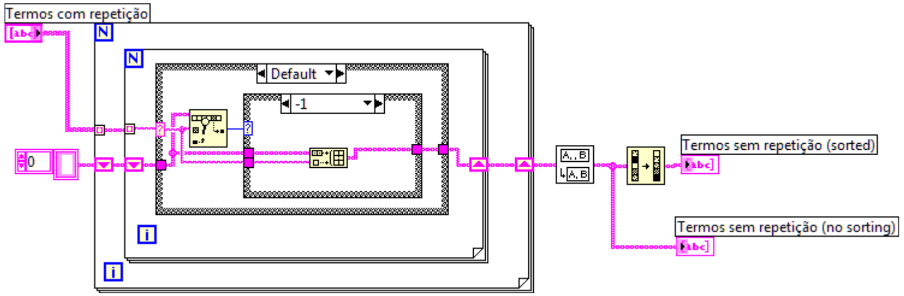

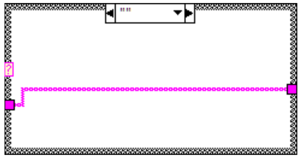
""

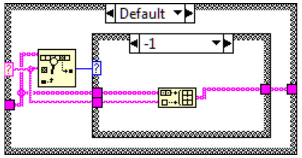
Default
-1

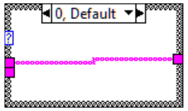
0, Default

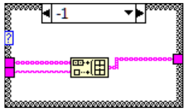
-1
