# Supplementary material for: Misdiagnosis and undiagnosis due to pattern similarity in Chinese medicine: a stochastic simulation study using pattern differentiation algorithm
Source: Chin Med. 2011 Jan 12;6:1. doi: 10.1186/1749-8546-6-1 (PMC3037949; doi:10.1186/1749-8546-6-1)

Pattern Differentiation Algorithm - PDA.vi

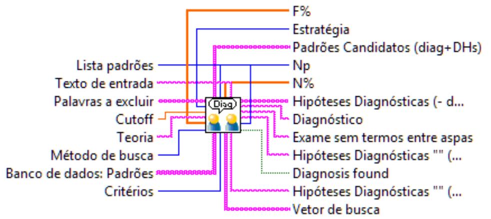

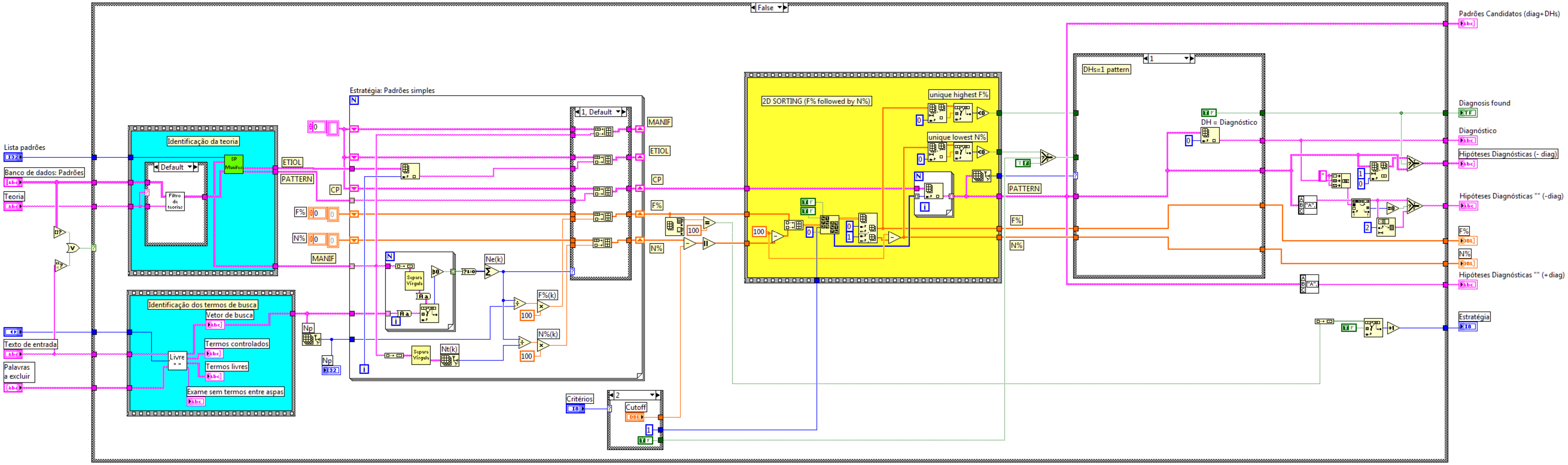

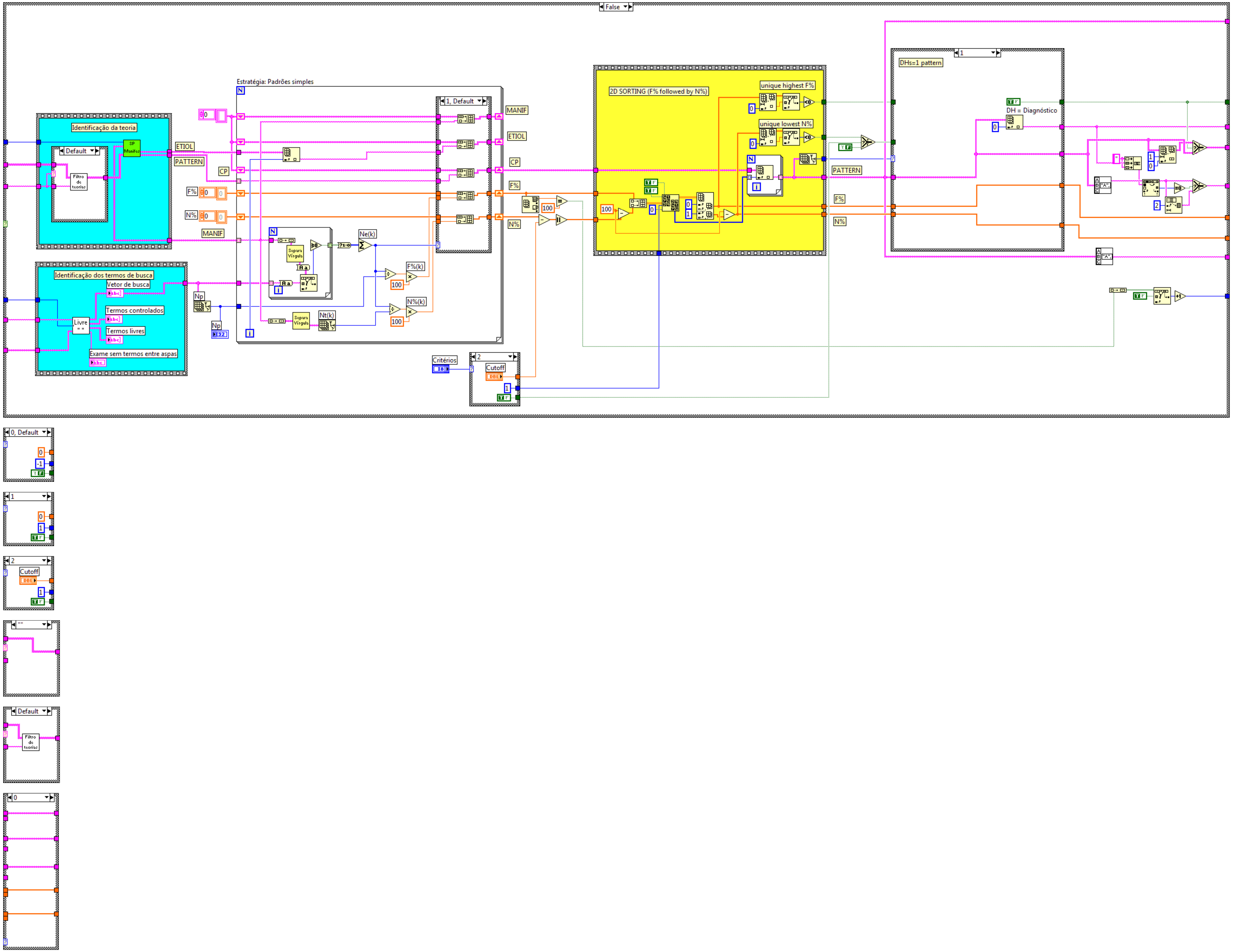
False
Identificação da teoria
Default
Filtro de teorias
ETIOL
PATTERN
CP
F%
N%
MANIF
Identificação dos termos de busca
Vetor de busca
Termos controlados
Termos livres
Exame sem termos entre aspas
Livre
Np
Estratégia: Padrões simples
1, Default
Ne(k)
F%(k)
N%(k)
Nt(k)
Separa Vírgula
100
Critérios
Cutoff
2D SORTING (F% followed by N%)
unique highest F%
unique lowest N%
DHs=1 pattern
DH = Diagnóstico
0, Default
1
2
0

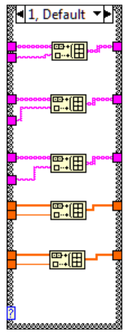
1, Default

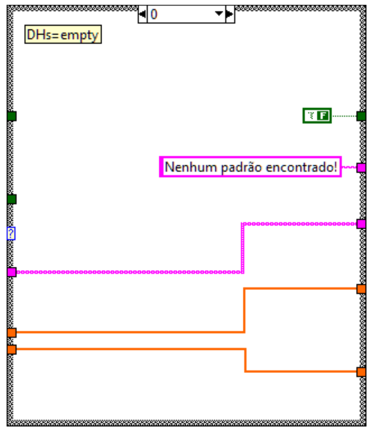
0
DHs=empty
Nenhum padrão encontrado!

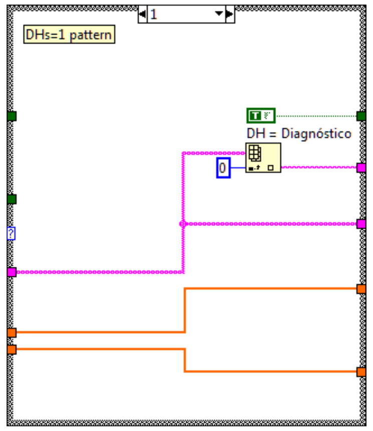
1
DHs=1 pattern
DH = Diagnóstico
0

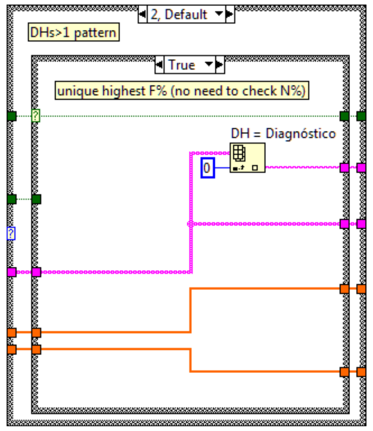
2, Default
DHs>1 pattern
True
unique highest F% (no need to check N%)
DH = Diagnóstico
0

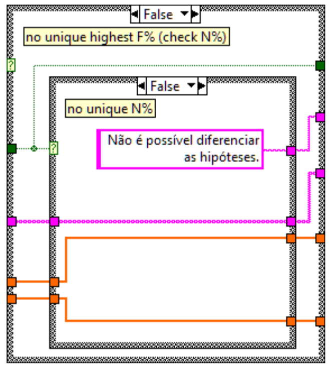
False
no unique highest F% (check N%)
False
no unique N%
Não é possível diferenciar as hipóteses.

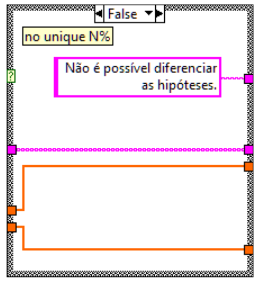
False
no unique N%
Não é possível diferenciar as hipóteses.

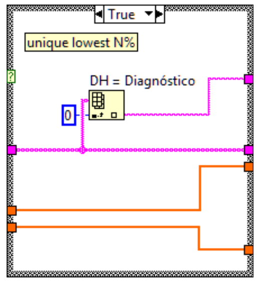
True
unique lowest N%
DH = Diagnóstico
0

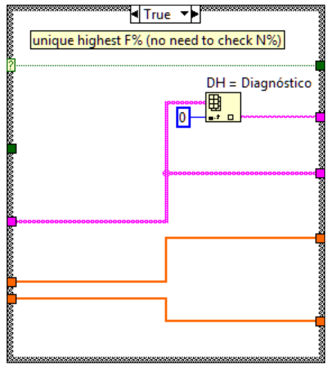
True
unique highest F% (no need to check N%)
DH = Diagnóstico
0

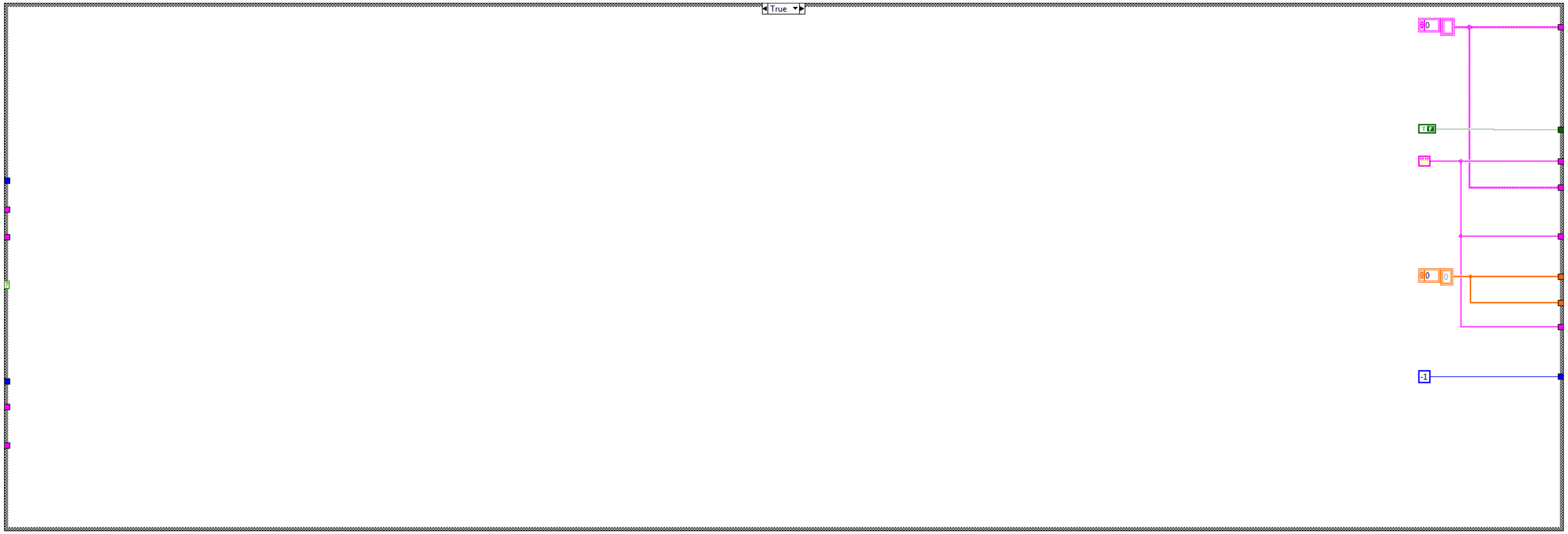

Busca de termos livre e aspas.vi

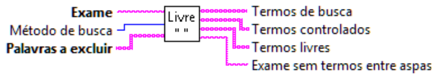

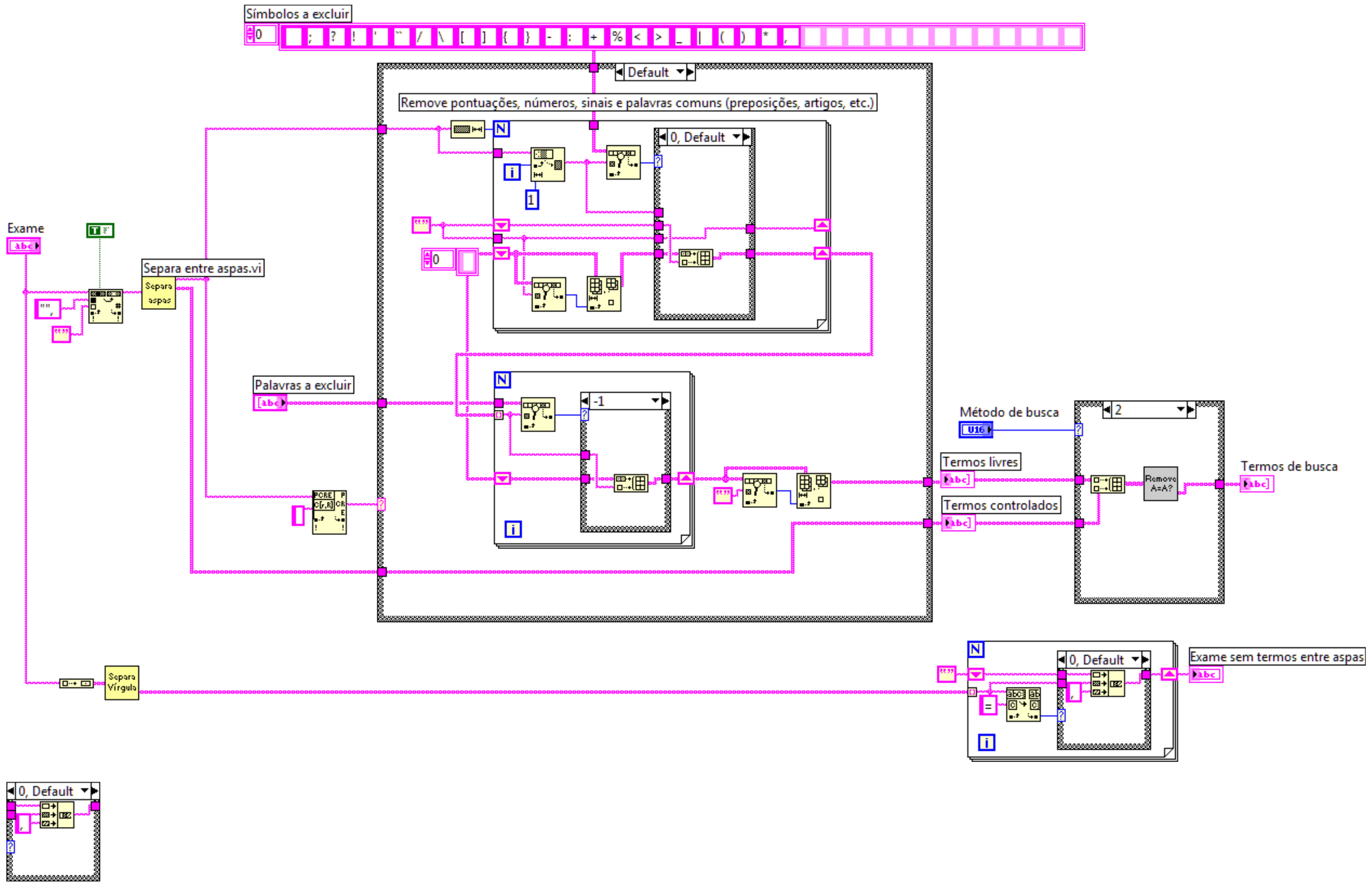

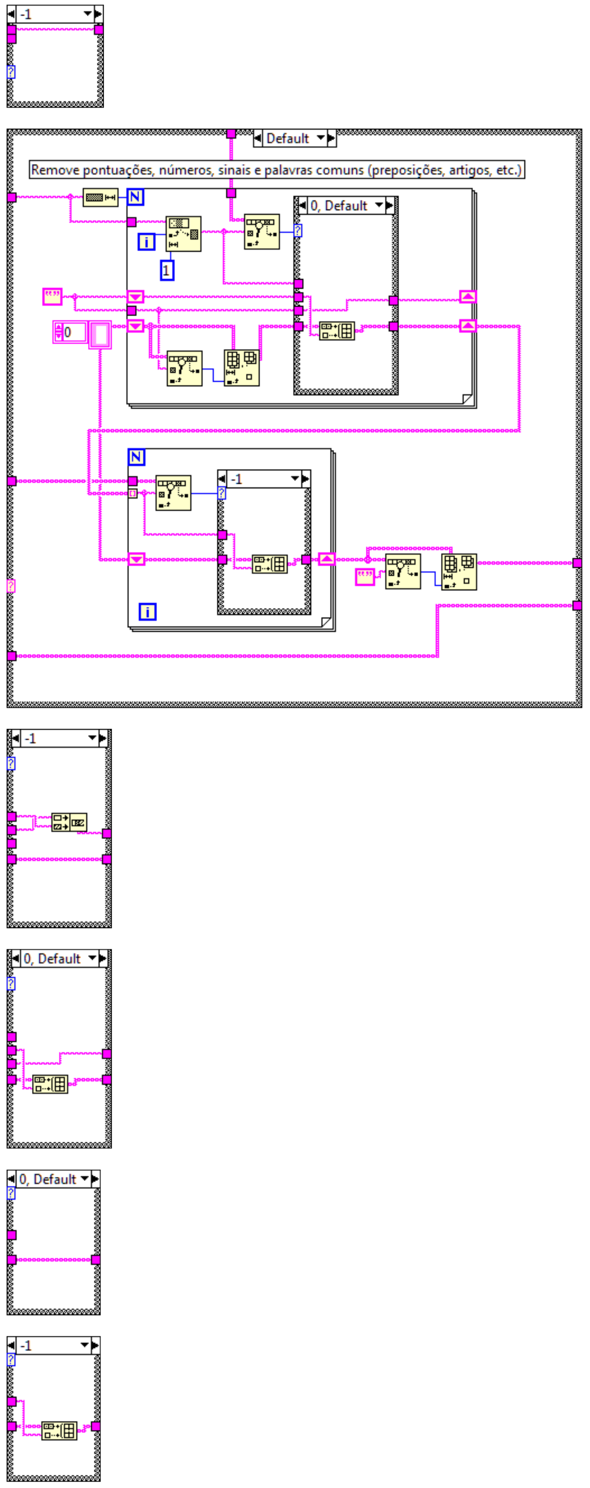
-1
Default
Remove pontuações, números, sinais e palavras comuns (preposições, artigos, etc.)
0, Default
-1
-1
0, Default
0, Default
-1

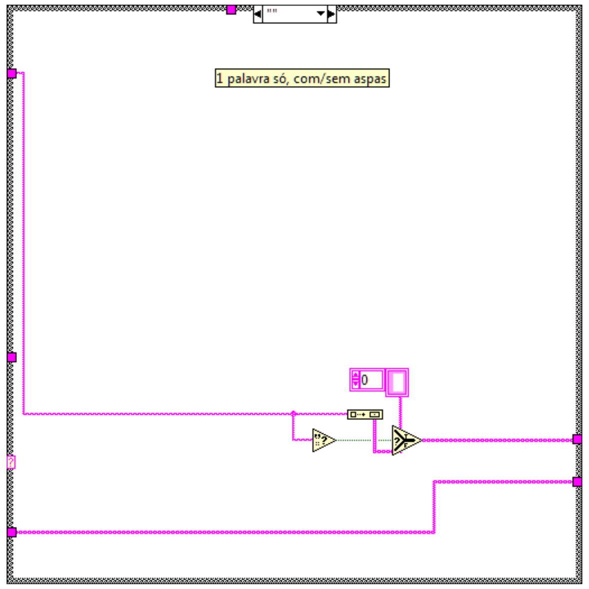

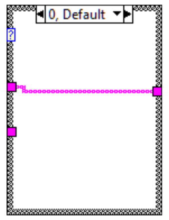

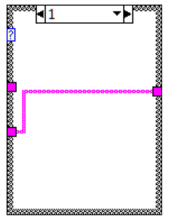

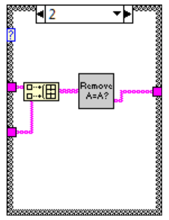

Separa entre vírgulas.vi

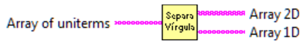

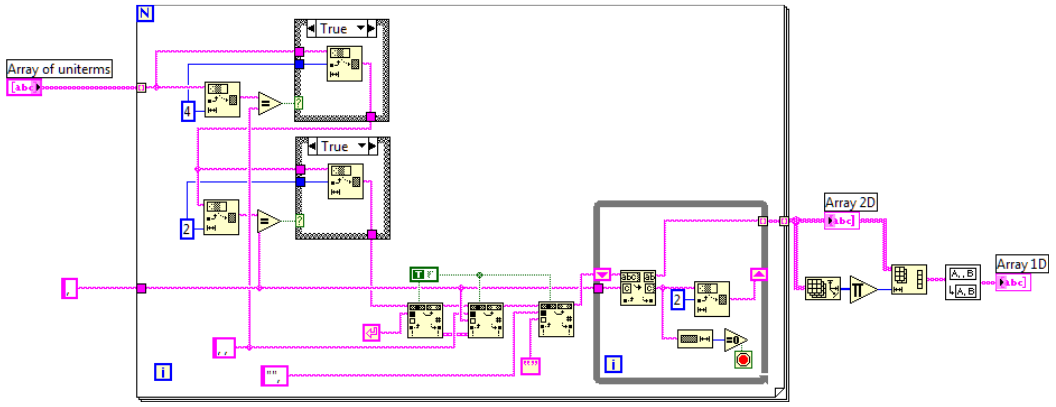

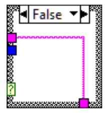

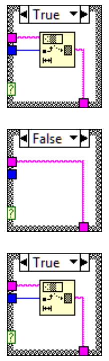

Remove espaços em branco.vi

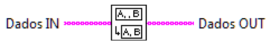

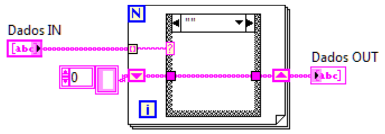

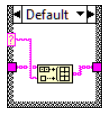

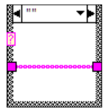

Separa entre aspas.vi

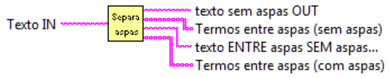

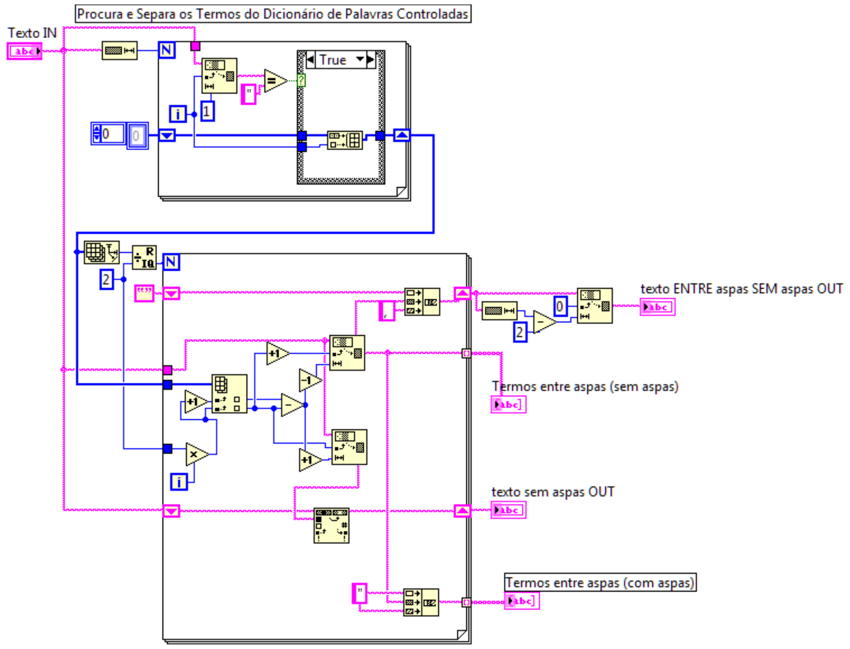

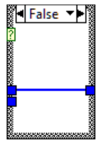

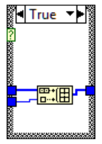

Remove termos repetidos.vi

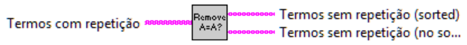

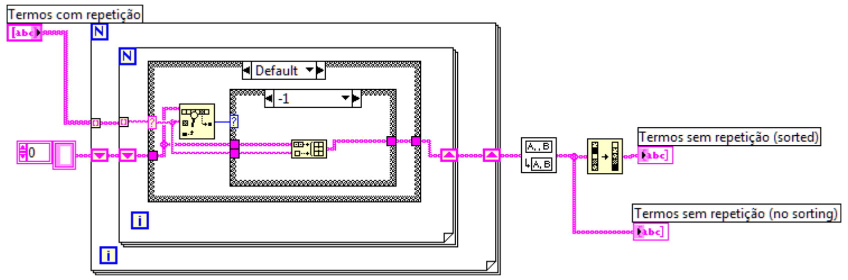

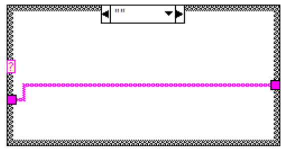

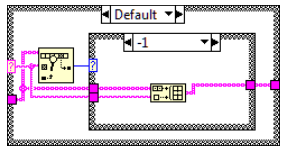

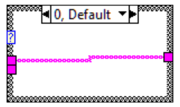

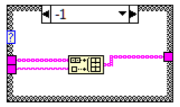

Filtro de padrões por teoria.vi

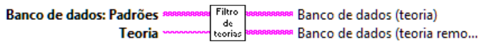

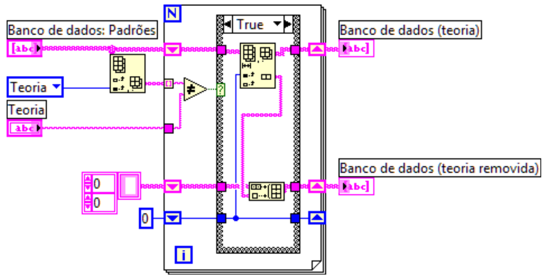

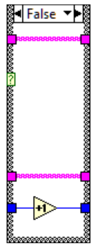

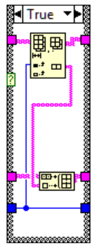

SP - Manifestações.vi

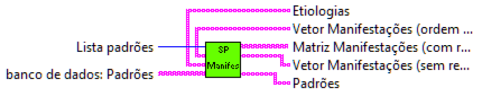

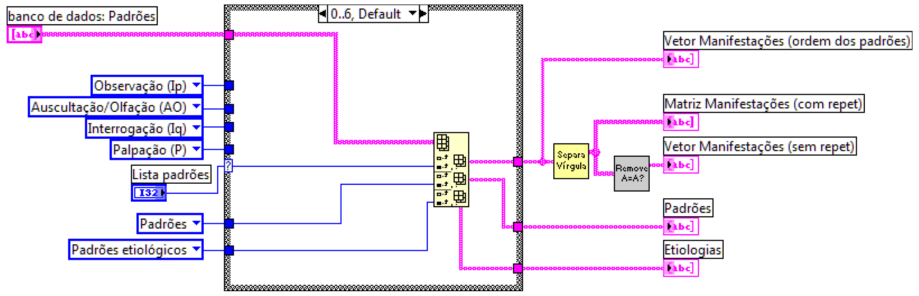

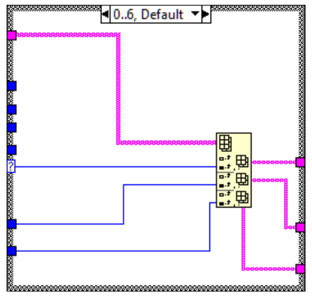

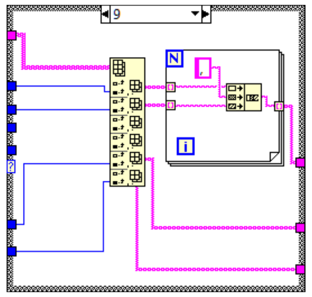

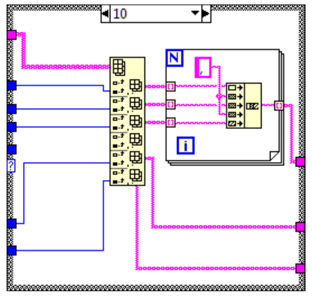

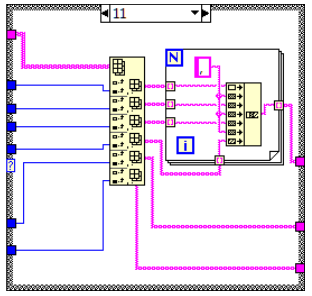

Sort 2D Array (numeric).vi

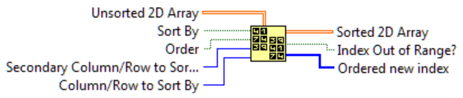

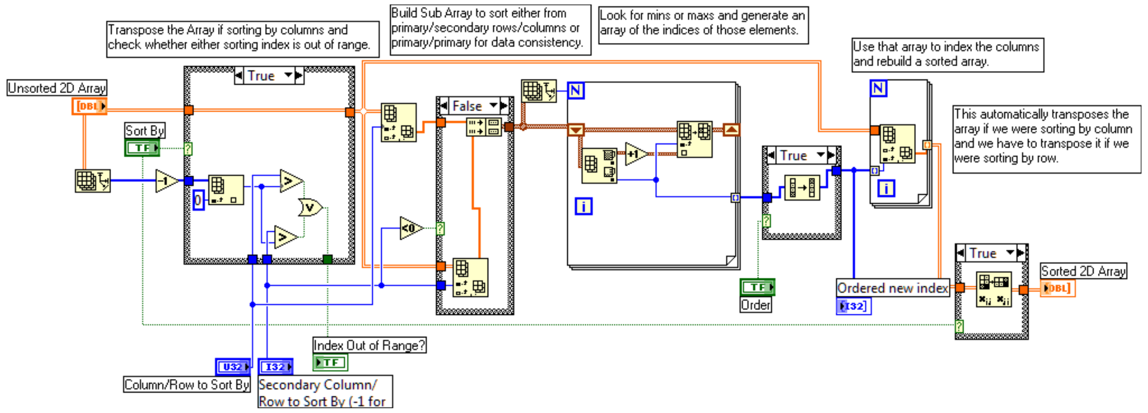

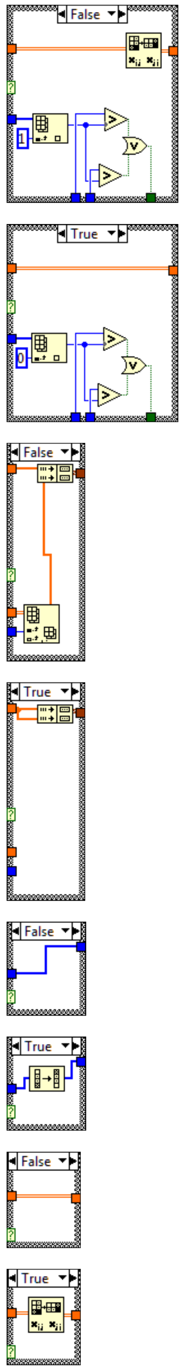

Agrupa entre aspas e vírgula.vi

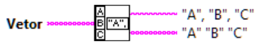

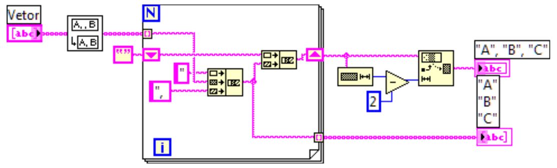

Supplement: Additional file 3 — Pattern differentiation algorithm. This file presents screenshots with the source code of the algorithms for pattern differentiation. [file 1749-8546-6-1-S3.PDF]
